# Adjacent segment disease after minimally invasive transforaminal lumbar interbody fusion for degenerative lumbar diseases: incidence and risk factors

**DOI:** 10.1186/s12891-022-05905-6

**Published:** 2022-11-14

**Authors:** Chao Yuan, Jing Zhou, Liran Wang, Zhongliang Deng

**Affiliations:** 1 Department of Orthopaedics, The Second Affiliated Hospital of Chongqing Medical University, 76 Linjiang Road, Yuzhong District, Chongqing, 400010 China; 2 Department of Orthopaedic Surgery, Affiliated Xinqiao Hospital, Army Medical University, No.183, Xinqiao Street, Shapingba District, Chongqing, 400037 People’s Republic of China

**Keywords:** Adjacent segment disease, Minimally invasive transforaminal lumbar interbody fusion, Degenerative lumbar diseases, Risk factors

## Abstract

**Study design:**

Retrospective study.

**Objectives:**

To explore the incidence and risk factors for symptomatic adjacent segment disease (ASD) in patients enveloped in degenerative lumbar diseases after minimally invasive transforaminal lumbar interbody fusion (MIS-TLIF).

**Methods:**

Data were retrospectively analyzed on 744 patients who underwent MIS-TLIF for degenerative lumbar diseases in our hospital from October 2012 to December 2018. The patients were divided into the ASD group and non-ASD (N-ASD) group on the basis of developing ASD at follow-up, and then the incidence of ASD was calculated. Clinical and radiological risk factors were assessed over time to determine their association with ASD by excluding less important factors.

**Results:**

Data were missing for 26 patients, while a total of 718 patients were successfully monitored after MIS-TLIF. Of the 718 individuals participated in the study, 34 (4.7%) patients plagued by ASD required surgical intervention. The average onset time of ASD was 62.7 ± 15.1 months. Univariate analysis results shows that age, bone mineral density (BMD), body mass index (BMI), preoperative adjacent intervertebral disc height and preoperative adjacent segment disc degeneration were significantly different between the ASD and N-ASD groups (*p* < 0.05). The logistic regression analysis results demonstrated that BMD (*p* = 0.039, OR = 0.986, 95% CI 0.899–1.115), BMI (*p* = 0.041, OR = 1.119, 95% CI 1.103–2.397), and preoperative adjacent intervertebral disc degeneration (*p* = 0.023, OR = 1.215, 95% CI 1.015–1.986) may be seen as risk factors for ASD after MIS-TLIF.

**Conclusions:**

The incidence of ASD was about 4.7% in patients suffer from degenerative lumbar diseases after MIS-TLIF. BMD, BMI and preoperative adjacent intervertebral disc degeneration might be the risk factors for the occurrence of ASD after MIS-TLIF. Our research also suggested that patients with lower BMD, higher BMI and disc preoperative adjacent segment disc degeneration were more likely to develop ASD after MIS-TLIF.

## Introduction

Degenerative lumbar diseases is a pathologic process that affects a substantial portion of aging population; with the acceleration of demographic aging, the incidence of degenerative lumbar diseases is on the rise [1]. When conservative measures fail, often surgical intervention is required. A minimally invasive transforaminal lumbar interbody fusion (MIS-TLIF) technique has been reported to reduce the iatrogenic soft tissue injury that occurs with muscle stripping and retraction during routine spinal exposure [2, 3]. Given the potential advantages of MIS-TLIF procedures surgery over the traditional open approach, including decreased blood loss, less soft tissue injury, shorter hospital length of stay, earlier recovery, and fewer complications, MIS-TLIF was increasingly being used to treat degenerative lumbar diseases [4, 5].

Although most patients experience symptomatic relief and improved quality of life after MIS-TLIF which provides adequate nerve decompression and stabilization through intersomatic fusion, but the biomechanical changes in the spine caused by fusion may accelerate the degenerative change of the adjacent unfused segments. Despite improvements in the efficacy of spinal fusion procedures, postoperative complications including revisions are still a dilemma to be solved urgently [6]. Adjacent segment disease (ASD) is a potential long-term complication and is one of the common reasons for revision surgery after lumbar fusion [7, 8]. ASD refers to degenerative changes in the unfused segment adjacent to the fusion segments after lumbar fusion, which may lead to the recurrence of symptoms. Despite different definitions, a high incidence of ASD after lumbar fusion has been reported, ranging from 5 to 49% [9, 10]. At present, ASD has become the main concern after lumbar fusion surgery, and a variety of risk factors related to ASD have been reported [11]. However, the exact pathogenesis of ASD is still unclear and few studies have focused on risk factors for ASD after MIS-TLIF, which makes our study more meaningful. In consideration of the increase during the lumbar fusion procedures, it is worthwhile to elucidate the etiology and risk factors of ASD. The aim of this study was to investigate the incidence and risk factors of ASD in patients with lumbar degenerative diseases after MIS-TLIF, so as to guide spinal surgeons in preoperative planning and reduce the incidence of ASD.

## Methods

### Patients

A retrospective analysis of prospectively collected data was performed on patients receiving MIS-TLIF at a single institution. A total of 744 patients who underwent MIS-TLIF were enrolled from the Second Affiliated Hospital of Chongqing Medical University. The inclusion criteria for this study were: [1] low-grade degenerative spondylolisthesis or isthmic spondylolisthesis according to Meyerding classification, [2] lumbar instability, [3] lumbar spinal stenosis, [4] patients with chronic lower back pain and/or leg pain unresponsive to conservative therapy for at least 3 months [5]. follow-up time ≥ 24 months; and ≤ 2 fusion segments.

**Fig. 1:**
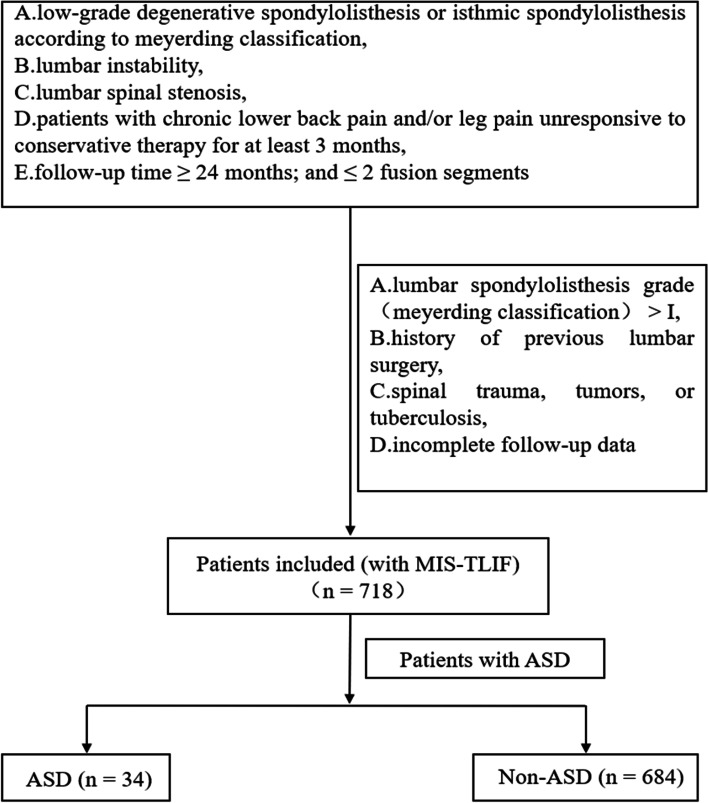
The specific inclusion and exclusion process and grouping

The exclusion criteria were as follows: [1] lumbar spondylolisthesis grade (Meyerding classification) > I, [2] history of previous lumbar surgery, [3] spinal trauma, tumors, or tuberculosis, [4] incomplete follow-up data. In this study, ASD was defined as a symptomatic including radiculopathy, stenosis, and instability in an adjacent segment after MIS-TLIF, for which a second operation was performed on the adjacent fusion segment [12]. The specific inclusion and exclusion process and grouping are shown in Fig. 1.

### Surgical procedures

All patients were treated with a Quadrant Retractor System (Medtronic Sofamor Danek, Memphis, TN, USA) while under general anesthesia and lying prone on a light transmission frame in accordance with the standard procedure described previously [4]. Briefly, unilateral paramedian skin incision is used (3–5 cm) for exposing facet joints，a tubular retractor was then introduced to the facet joint and part of the vertebral lamina and superior and inferior articular processes on the symptomatic side, and fully decompress the central canal and lateral recess. The cartilage endplate of the intervertebral space was prepared, the bone that was removed during the operation was trimmed into broken bone particles and used to fill the fusion cage. A polyether-etherketone (PEEK) cage filled with autologous bone graft was inserted obliquely across the discs pace. The nerves were then examined to ensure they were adequately decompressed. After the retractor was removed, the percutaneous pedicle screw system was placed through the minute incision under fluoroscopic guidance.

### Clinical and radiological evaluation

In line with the previous reports, potential risk factors for ASD development we have selected and evaluated, including age, gender, smoking history, diabetes mellitus, hypertension, BMI, BMD, operation time, postoperative blood loss, preoperative adjacent disc degeneration, preoperative adjacent disc height, etc. [13, 14]. The T score obtained from the lumbar Dual Energy X-ray Absortiometry (DEXA, GE Lunar; Prodigy, Madison, WI) scans were used to assess the association between preoperative overall fracture risk and BMD before surgery. Preoperative adjacent disc degeneration was evaluated at the instance of the classification of Pfirrmann (I–V) depend on MRI of patients [15]. Patients were divided into ASD group and N-ASD group according to the presence or absence of ASD at the last follow-up.

Imaging data were collected and analysed at the imaging department of our hospital. Demographic information was retrieved from our hospital’s data base of follow-up findings.

### Statistical analyses

Statistical analyses were performed using SPSS Version 23.0 (SPSS, Inc., Chicago, Illinois, USA). All data were presented as the mean ± standard deviation. The independent t-test, rank-sum test and χ2 test were used to perform single-factor correlation analysis on various variables to find potential risk factors for ASD. The potential risk factors were further analyzed by logistic regression to identify the independent risk factors for the occurrence of ASD after MIS-TLIF. Candidate variables with *p* < 0.1 were selected for multivariate logistic regression, and then the Odds ratio (OR) with their 95% confidence interval were calculated. *p* values < 0.05 were accepted for significance.

## Results

Patients treated with MIS-TLIF between October 2012 and December 2018 were monitored. Ultimately, 744 screened patients underwent at least 24 months of follow-up (mean duration, 34.3 ± 14.7 months), while the data of 26 patients were missing. All patients had to have been treated conservatively for at least 3 months without success before considering a surgical intervention. A total of 718 (386 male and 332 female, mean age at surgery, 56.3 ± 13.7 years; age range, 24-81 years) patients who underwent MIS-TLIF met the inclusion criteria for the study were identified. Among them, there were 634 cases of single-segment fusion and 84 cases of two-segment fusion. The average number of fusion segments was 1.1 ± 0.3 (Table 1).

**Table 1:** Characteristics of the patients

Item	MIS-TLIF
Number of patients	*n* = 718
Mean age (y)	56.3 ± 13.7 (24–81)
Gender (M/F)	386/332
Diagnoses (n)	–
Degenerative spondylolisthesis	270
Isthmic spondylolisthesis	198
Lumbar instability	67
Lumbar spinal stenosis	183
Fusion segments (n)	–
One-level	634
Two-level	84
Average segments	1.1 ± 0.3
Follow-up period (mo.)	34.3 ± 14.7 (24–106)

Of the 718 patients, 34 (19 male and 15 female) developed ASD and underwent reoperation. The incidence of ASD was 4.7%, and the average onset time of ASD was 62.7 ± 15.1 months after MIS-TLIF. The segment of the previous operation inpatients with ASD was as follows: L3–L4, *n* = 5; L3–L5, *n* = 6; L4–L5, *n* = 5; L5–S1, *n* = 18. The ASD segment was as follows: L2/3, *n* = 1; L4/5, *n* = 22; L5/S1, *n* = 11. Of those patients, 23 patients underwent surgery for the treatment of upper ASD, 11 patients for lower ASD (Table 2). Among the patients with ASD, 28 patients underwent the decompression and fusion in their reoperation, 6 patients underwent simple decompression (Table 3). The symptoms of all ASD patients were alleviated, and the function was improved after reoperation.

**Table 2:** Characteristics of the patients with ASD

Item	ASD
Number of patients	*n* = 34
Mean age (y)	61.3 ± 11.4
Gender(M/F)	19/15
Follow-up period (mo.)	62.7 ± 15.1
Primary surgery segment	–
L3–L4	5
L3–L5	6
L4–L5	5
L5–S1	18
ASD position (n)	–
Cranial	23
Caudal	11
ASD segment (n)	–
L2/3	1
L4/5	22
L5/S1	11

**Table 3:** Description of reoperation in patients with ASD

ASD surgical strategy	Number of patients
MIS-TLIF	4
OLIF	13
TLIF	11
PELD	6
Total	34

*OLIF* oblique lumbar interbody fusion, *TLIF* transforaminal lumbar interbody fusion, *PELD* percutaneous endoscopic lumbar discectomy

Univariate analysis suggested that age, BMD, BMI, preoperative adjacent intervertebral disc height and preoperative adjacent segment disc degeneration were significantly different between the ASD group and N-ASD group (*p* < 0.05) (Tables 4 and 5). The logistic regression analysis demonstrated that BMD (*p* = 0.039, OR = 0.986, 95% CI 0.899–1.115), BMI (*p* = 0.041, OR = 1.119, 95% CI 1.103–2.397) and preoperative adjacent intervertebral disc degeneration (*p* = 0.023, OR = 1.215, 95% CI 1.015–1.986) can be considered as the risk factors for emergence of ASD when patients underwent MIS- TLIF.

**Table 4:** Characteristics of patients with or without ASD after MIS-TLIF

Variable	ASD *n* = 34	N-ASD *n* = 684	***p*** value
Age (y)	64.7 ± 15.1	55.1 ± 14.6	**0.039**
Gender (M/F)	22/12	324/360	0.348
Smoking (yes/no)	8/26	141/543	0.682
Presence of diabetes (yes/no)	5/29	74/610	0.480
Presence of hypertension (yes/no)	10/24	234/450	0.564
Bone mineral density (T score)	−2.8 ± 1.4	−1.7 ± 0.8	**0.038**
BMI (kg/m^2^)	29.7 ± 2.1	22.4 ± 2.1	**0.040**
Operative time (minutes)	108.7 ± 12.1	110.3 ± 17.2	0.780
Blood loss (ml)	120.3 ± 20.6	113.8 ± 16.9	0.521

**Table 5:** Comparison of radiological variables between the ASD Group and N-ASD Group

Variable	ASD (*n* = 34)	N-ASD (*n* = 684)	***p*** value
Preoperative adjacent disc height (mm)	8.1 ± 1.2	11.9 ± 1.6	**0.043**
Preoperative adjacent disc degeneration (Pfirrmann classification)	–	–	–
I	0	78	–
II	1	467	–
III	10	128	**0.015**
IV	18	11	–
V	5	0	–

Variables with *p* < 0.1 were further taken into account in the multivariate logistic regression analysis. Our multivariate analysis results suggested that BMD (*p* = 0.039, OR = 0.986, 95% CI 0.899–1.115), BMI (*p* = 0.041, OR = 1.119, 95% CI 1.103–2.397) and disc preoperative adjacent segment disc degeneration (*p* = 0.023, OR = 1.215, 95% CI 1.015–1.986) were independently associated with the development of ASD after MIS-TLIF (Table 6). Patients who had lower BMD, higher BMI and disc preoperative adjacent segment disc degeneration were more likely to develop ASD.

**Table 6:** Logistic regression analysis of risk factors in ASD patients

Variable	95% CI	Odds ratio (OR)	*P* value
Age	0.956–0.998	0.989	0.563
BMD	0.899–1.115	0.986	**0.039**
BMI	1.103–2.397	1.119	**0.041**
Preoperative adjacent disc height	0.946–1.109	0.982	0.669
Preoperative adjacent segment disc degeneration	1.015–1.986	1.215	**0.023**

## Discussion

Over the past decade, MIS-TLIF has been demonstrated efficacy in the treatment of degenerative lumbar diseases, but postoperative complications including ASD still pose a significant concern for surgeons [16]. ASD can be classified as symptomatic and radiographic [17], it was defined as a symptomatic including radiculopathy, stenosis, and instability in an adjacent segment after MIS-TLIF in our research, causing clinically significant symptoms and requiring reoperation. Furthermore, imaging findings of ASD, reoperation rate and risk factors were elaborated in our study. As mentioned above, imaging degeneration of adjacent segments after lumbar fusion is common and may not be related to the clinical symptoms of patients, which should be examined in combination with the clinical manifestations of patients [18].

**Fig. 2:**
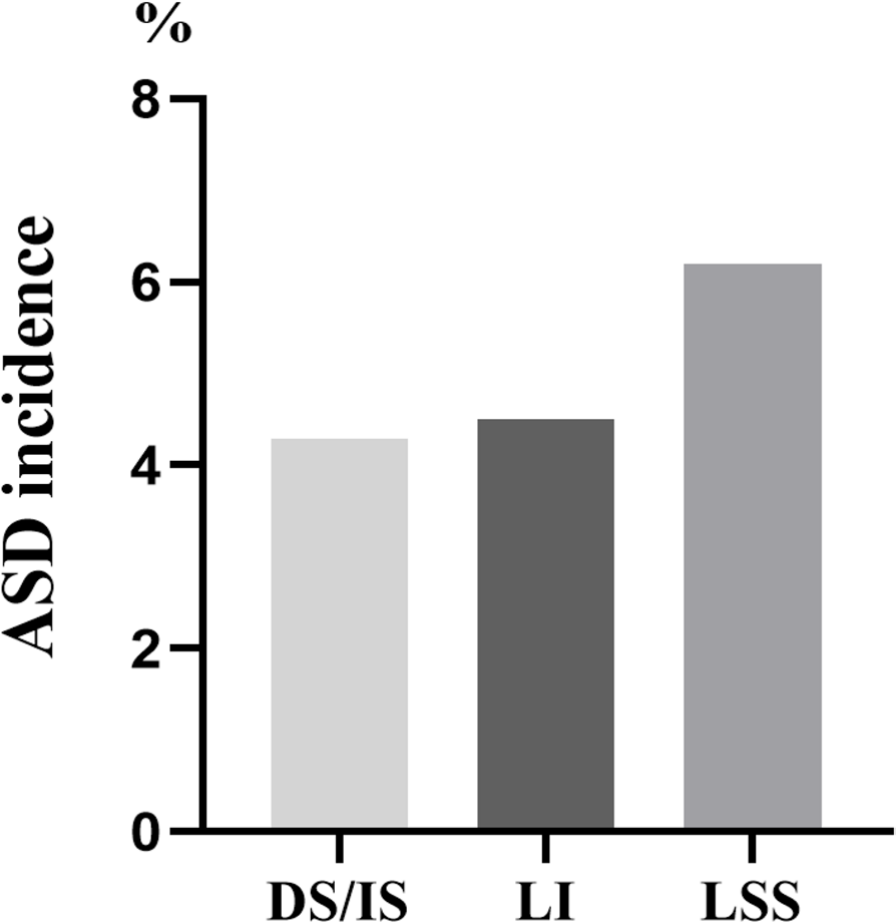
The incidence of ASD was not significantly different among the DS/IS, LI, LSS. DS: degenerative spondylolisthesis, IS: isthmic spondylolisthesis, LI: lumbar instability, LSS: lumbar spinal stenosis

In the present study, 4.7% of patients experienced ASD after MIS-TLIF, which is approximately consistent with the previously reported 3.9 to 14% reoperation rate of adjacent segments after lumbar fusion surgery [19, 20]. The lower incidence of our results may be attributed to the situation that the definition of ASD was confined to those cases of ASD that were severe enough to require reoperation. Additionally, in our research, we found ASD is more often involved in the cranial segment than the caudal segment, which is consistent with the findings of many studies [21, 22]. Chen et al. expounded that the cranial segment is more prone to ASD, which may be due to the increased pressure of the intervertebral disc in the adjacent segment after lumbar fusion and fixation, especially the higher pressure in the cranial segment than that in the tail segment [23]. Patients with incomplete imaging data or lost to follow-up were not considered. Therefore, the actual incidence of ASD is expected to increase with longer follow-up. In addition, the type of preoperative degenerative lumbar disease had no significant effect on the incidence of ASD were unfolded in our results (Fig. 2). The lower likelihood of selection bias may be due to smaller differences in the incidence of various degenerative lumbar diseases.

Authenticating the risk factors is one of the important measures to prevent the occurrence and development of ASD. The emerging of ASD after lumbar fusion may be affected by many factors, including age, gender, smoking history, diabetes mellitus, hypertension, BMI, BMD, preoperative adjacent disc degeneration, preoperative adjacent disc height, sagittal vertical axis (SVA) and long-segment fusion [11, 20]. The precise pathophysiology of ASD remains still unclear despite several studies investigating the mechanisms of pathogenesis [8]. It is now widely accepted that ASD caused by biomechanical changes and spinal fusion increases the pressure and motion of adjacent unfused segments and accelerates the degeneration of these segments [24]. Our multivariate analysis clearly identified that lower BMD, higher BMI and preoperative adjacent segment disc degeneration are independent risk factors for developing ASD after undergoing MIS-TLIF. Although some studies have suggested that smoking may be a trigger that affects intervertebral disc degeneration [25, 26], but the correlation between smoking and ASD has not been clearly confirmed in our results, which may be due to some limitations in our study or may indicate that there is no association between smoking and the emergence of ASD.

BMD is widely used to diagnose osteoporosis and assess effectiveness of treatments for osteoporosis by World Health Organization (WHO). Osteoporosis, which is prevalent in postmenopausal women or older people, was thought to be the inducement to ASD. The fact that ASD is more common in postmenopausal women suggests that osteoporosis may be a stimulator of ASD, which is consistent with the clinical findings of Etebar et al. [27]. Based on animal experiments, Zhou et al. suggested that osteoporosis could aggravate the development of ASD [28]. A study has revealed that osteoporosis may increase the risk of disc degeneration in the lumbar spine by adversely changing the loading pattern and intradiscal pressure [29]. When osteoporosis occurs, decreased bone mineral density can reduce the stiffness, influence the stress distribution, and lead to significant changes of the biomechanical in the adjacent segment [30]. In this presented study, we found ASD was associated with osteoporosis, which is consistent with findings of previous research [31]. However, some studies have reported an association between ASD and osteoporosis that needs to be further confirmed [32, 33]. The contradictory data may be a result of a difference in the degree of osteoporosis in patients in the different studies.

There was still controversy about whether there is an association between age and ASD. Some studies have indicated that age as a risk factor [19]，but Zhong et al. held the opposite view, and our multivariate analysis results also suggested no association between age and ASD [34]. Although age was not associated with ASD as confirmed by our study, spinal surgeons should still be aware of the possibility of an increased risk of ASD in older patients when considering lumbar fusion surgery.

BMI is an objective and simple indicator that is generally accepted. Higher BMI was concurrenced by the increased stress on the lumbar spine, which can accelerate disc degeneration caused by the increased burden to absorb the loading forces, leading to abnormal loading on surrounding facet joints, spinal ligaments, and paraspinal muscles [35]. Seyed et al. demonstrated that patients who had higher preoperative BMI present a statistically increase in the risk of developing ASD [36]. Wang and his colleagues reckoned that patients with higher preoperative BMI had a significantly increased risk of ASD after lumbar fusion surgery [7]. Furthermore, a study conducted by Zheng et al. also suppose that patients with higher BMI were more likely to develop ASD [37]. This is consistent with our findings, patients with higher BMI had a tendency to experience ASD. On the basis of the result mentioned above, we supposed that it is necessary to recommend appropriate weight loss for patients with higher BMI before and after surgery to reduce the incidence of ASD.

The correlation between the height of the intervertebral space and the degree of disc degeneration is rarely reported, but some studies suggest that collapse of the intervertebral space is often accompanied by severe degeneration of the intervertebral disc and the appearance of neurological symptoms [38]. While, our multivariate analysis showes that preoperative adjacent segment intervertebral height may be a protective factor against ASD (*p* = 0.669, OR = 0.982, 95% CI 0.946–1.109).

**Fig. 3:**
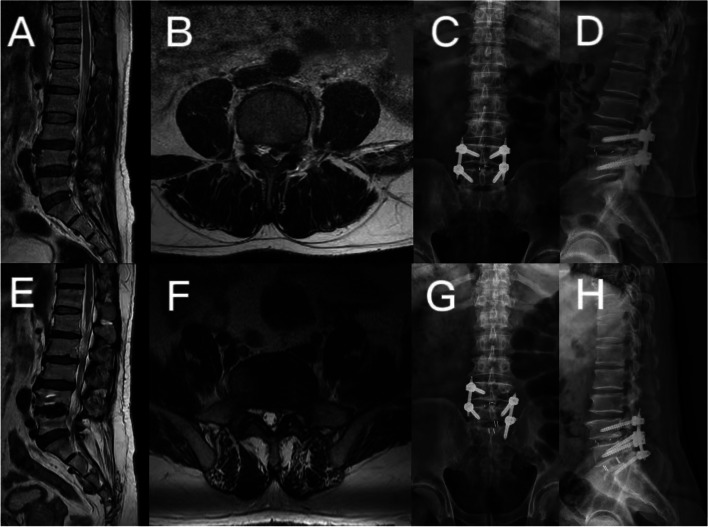
A 54-year-old male, with L4/5 lumbar disc herniation (**A** and **B**), MIS-TLIF was performed on L4/5 in November 2012 (**C** and **D**). In August 2021, the patient developed numbness and pain in the left leg. MRI showed that the L5/S1 disc was herniated and compress the left nerve root (**E** and **F**). His diagnosis of ASD and MIS-TLIF was performed by MRI at L5/S1 (**G** and **H**)

Postoperative adjacent segment degeneration tends to occur in patients with late preoperative disc degeneration [39, 40]. Consistently, preoperative intervertebral disc degeneration at adjacent segment was considered as a risk factor for the ASD after MIS-TLIF (*p* = 0.023, OR = 1.215, 95% CI 1.015–1.986) in our research. This conclusion is supported by Wang et al., who perceived that preoperative Pfirrmann classification of ≥3 was significantly associated with a higher incidence of ASD [8]. The reduced flexibility and increased stiffness of the lumbar spine caused by fixation and fusion lead to biomechanical changes in adjacent segments that accelerate postoperative degeneration of adjacent segments [41–43]. Therefore, considering these pathophysiological changes, preoperative adjacent segment degeneration may be positively correlated with the occurrence of ASD (Fig. 3).

There were some limitations in our study. This study was a retrospective and nonrandomized, there may be bias in the analysis. A randomized controlled trial should be considered in order to provide convincible evidence-based conclusions. Moreover, here may be some risk factors that are yet unknown, more studies need to be performed in the future.

## Conclusion

In this study, we retrospectively analyzed 718 patients who underwent MIS-TLIF, 34 of whom (4.7%) developed ASD and underwent resurgery. Our study also suggested that the patients who had lower BMD, higher BMI and disc preoperative adjacent segment disc degeneration were more likely to develop ASD after MIS-TLIF.

## Data Availability

The datasets used and/or analysed during the current study are available from the corresponding author on reasonable request.

## References

[1] Vos T, Flaxman AD, Naghavi M, et al. Years lived with disability (YLDs) for 1160 sequelae of 289 diseases and injuries 1990-2010: a systematic analysis for the Global Burden of Disease Study 2010 [published correction appears in Lancet. 2013;381(9867):628. AlMazroa, Mohammad A [added]; Memish, Ziad A [added]]. Lancet. 2012;380(9859):2163–96. 10.1016/S0140-6736(12)61729-2 PMC6350784 23245607

[2] Seldomridge JA, Phillips FM (2010). Minimally invasive spine surgery. Minim Invasive Neurosurg.

[3] Kim C W. Scientific basis of minimally invasive spine surgery: prevention of multifidus muscle injury during posterior lumbar surgery. Spine. 2010;35(26 Suppl):S281. 10.1097/BRS.0b013e3182022d32 21160391

[4] Jian W, Yue Z, Zheng FZ (2011). Minimally invasive or open transforaminal lumbar interbody fusion as revision surgery for patients previously treated by open discectomy and decompression of the lumbar spine. Eur Spine J.

[5] Tian W, Xu YF, Liu B, et al. Computer-assisted Minimally Invasive Transforaminal Lumbar Interbody Fusion May Be Better Than Open Surgery for Treating Degenerative Lumbar Disease. Clin Spine Surg. 2017;30(6):237–42. 10.1097/BSD.0000000000000165 28632545

[6] Charosky S, Guigui P, Blamoutier A (2012). Complications and risk factors of primary adult scoliosis surgery: a multicenter study of 306 patients. Spine.

[7] Wang H, Ma L, Yang D (2017). Incidence and risk factors of adjacent segment disease following posterior decompression and instrumented fusion for degenerative lumbar disorders. Medicine.

[8] Wang T, Ding W (2020). Risk factors for adjacent segment degeneration after posterior lumbar fusion surgery in treatment for degenerative lumbar disorders: a meta-analysis. J Orthop Surg Res.

[9] Bagheri SR, Alimohammadi E, Zamani Froushani A, Abdi A. Adjacent segment disease after posterior lumbar instrumentation surgery for degenerative disease: Incidence and risk factors. J Orthop Surg (Hong Kong). 2019;27(2):2309499019842378. 10.1177/2309499019842378. 10.1177/2309499019842378 31046589

[10] Okuda S, Nagamoto Y, Matsumoto T, Sugiura T, Takahashi Y, Iwasaki M. Adjacent Segment Disease After Single Segment Posterior Lumbar Interbody Fusion for Degenerative Spondylolisthesis: Minimum 10 Years Followup. Spine (Phila Pa 1976). 2018;43(23):E1384–8. 10.1097/BRS.0000000000002710 29794583

[11] Burch M, Wiegers N, Patil S, et al. Incidence and risk factors of reoperation in patients with adjacent segment disease: A meta-analysis. J Craniovertebral Junction Spine; 11(1):9–16. 10.4103/jcvjs.JCVJS_10_20 PMC7274364 32549706

[12] Hilibrand AS, et al. Adjacent segment degeneration and adjacent segment disease: the consequences of spinal fusion. Spine J. 2004;4(6 Suppl):190S–4S. 10.1016/j.spinee.2004.07.007. 10.1016/j.spinee.2004.07.007 15541666

[13] Lau KKL, Samartzis D, To NSC, Harada GK, An HS, Wong AYL (2021). Demographic, surgical, and radiographic risk factors for symptomatic adjacent segment disease after lumbar fusion: a systematic review and Meta-analysis. J Bone Joint Surg Am.

[14] Mesregah MK, Yoshida B, Lashkari N (2022). Demographic, clinical, and operative risk factors associated with postoperative adjacent segment disease in patients undergoing lumbar spine fusions: a systematic review and meta-analysis. Spine J.

[15] Pfirrmann CW, Metzdorf A, Zanetti M, Hodler J, Boos N. Magnetic resonance classification of lumbar intervertebral disc degeneration. Spine (Phila Pa 1976). 2001;26(17):1873–8. 10.1097/00007632-200109010-00011 11568697

[16] Yang Y, Liu B, Rong LM (2015). Microendoscopy-assisted minimally invasive transforaminal lumbar interbody fusion for lumbar degenerative disease: short-term and medium-term outcomes. Int J Clin Experiment Med.

[17] Hikata T, Kamata M, Furukawa M. Risk factors for adjacent segment disease after posterior lumbar interbody fusion and efficacy of simultaneous decompression surgery for symptomatic adjacent segment disease. J Spinal Disord Tech. 2014;27(2):70–5. 10.1097/BSD.0b013e31824e5292 22460400

[18] Abraham E, Manson N, Mckeon M (2014). The incidence of adjacent segment breakdown in Polysegmental thoracolumbar fusions of three or more levels with minimum 5-year follow-up. Global Spine J.

[19] Alentado VJ, Lubelski D, Healy AT, et al. Predisposing Characteristics of Adjacent Segment Disease After Lumbar Fusion. Spine (Phila Pa 1976). 2016;41(14):1167–72. 10.1097/BRS.0000000000001493 26863261

[20] Saavedra-Pozo FM, Deusdara RAM, Benzel EC (2014). Adjacent segment disease perspective and review of the literature. Ochsner J.

[21] F Pellisé, A Hernández, Vidal X, et al. Radiologic assessment of all unfused lumbar segments 7.5 years after instrumented posterior spinal fusion. Spine. 2007;32(5):574. 10.1097/01.brs.0000256875.17765.e6 17334293

[22] Rahm MD, Hall BB (1996). Adjacent-segment degeneration after lumbar fusion with instrumentation: a retrospective study. J Spinal Disord.

[23] Chen WJ, Lai PL, Niu CC (2001). Surgical treatment of adjacent instability after lumbar spine fusion. Spine.

[24] Kumar MN, Jacquot F, Hall H (2001). Long-term follow-up of functional outcomes and radiographic changes at adjacent levels following lumbar spine fusion for degenerative disc disease. Eur Spine J.

[25] Ogawa T, Matsuzaki H, Uei H, Nakajima S, Tokuhashi Y, Esumi M (2005). Alteration of gene expression in intervertebral disc degeneration of passive cigarette- smoking rats: separate quantitation in separated nucleus pulposus and annulus fibrosus. Pathobiology..

[26] Tu TH, Kuo CH, Huang WC, Fay LY, Cheng H, Wu JC (2019). Effects of smoking on cervical disc arthroplasty. J Neurosurg Spine.

[27] Etebar S, Cahill DW (1999). Risk factors for adjacent-segment failure following lumbar fixation with rigid instrumentation for degenerative instability. J Neurosurg.

[28] Zhou Z, Tian FM, Wang P (2015). Alendronate prevents intervertebral disc degeneration adjacent to a lumbar fusion in Ovariectomized rats. Spine.

[29] Tsouknidas A, Sarigiannidis SO, Anagnostidis K, Michailidis N, Ahuja S. Assessment of stress patterns on a spinal motion segment in healthy versus osteoporotic bony models with or without disc degeneration: a finite element analysis. Spine J. 2015;15(3 Suppl):S17–22. 10.1016/j.spinee.2014.12.148 25576902

[30] Park P, Garton HJ, Gala VC (2004). Adjacent segment disease after lumbar or lumbosacral fusion: review of the literature. Spine.

[31] Ankrah NK, Eli IM, Magge SN, Whitmore RG, Yew AY (2021). Age, body mass index, and osteoporosis are more predictive than imaging for adjacent-segment reoperation after lumbar fusion. Surg Neurol Int.

[32] Reza S, Bagheri E, et al. Adjacent segment disease after posterior lumbar instrumentation surgery for degenerative disease: incidence and risk factors. J Orthopaedic Surg. 2019. 10.1177/2309499019842378 31046589

[33] Li J, Xu W, Zhang X, Xi Z, Xie L. Biomechanical role of osteoporosis affects the incidence of adjacent segment disease after percutaneous transforaminal endoscopic discectomy. J Orthop Surg Res. 2019;14(1):131. Published 2019 May 14. 10.1186/s13018-019-1166-1. 10.1186/s13018-019-1166-1 PMC6515674 31088476

[34] Zhong ZM, Deviren V, Tay B (2017). Adjacent segment disease after instrumented fusion for adult lumbar spondylolisthesis: incidence and risk factors. Clin Neurol Neurosurg.

[35] Wang H, Ma L, Yang D, Wang T, Yang S, Wang Y, et al. Incidence and risk factors for the progression of proximal junctional kyphosis in degenerative lumbar scoliosis following long instrumented posterior spinal fusion. Medicine (Baltimore). 2016;95(32):e4443. 10.1097/MD.0000000000004443 PMC4985315 27512860

[36] Bagheri SR, et al. Adjacent segment disease after posterior lumbar instrumentation surgery for degenerative disease: incidence and risk factors. J Orthop Surg. 2019. 10.1177/2309499019842378 31046589

[37] Zheng G, Wang C, Wang T, et al. Relationship between postoperative lordosis distribution index and adjacent segment disease following L4-S1 posterior lumbar interbody fusion. J Orthop Surg Res. 2020;15(1):129. 10.1186/s13018-020-01630-9 PMC7119009 32245387

[38] Berlemann U, Gries NC, Moore RJ (1998). The relationship between height, shape and histological changes in early degeneration of the lower lumbar discs. Eur Spine J.

[39] Anandjiwala J, Seo JY, Ha KY (2011). Adjacent segment degeneration after instrumented posterolateral lumbar fusion: a prospective cohort study with a minimum five-year follow-up. Eur Spine J.

[40] Kim JY, Paik H K, et al. Paraspinal muscle, facet joint, and disc problems: risk factors for adjacent segment degeneration after lumbar fusion. Spine J, 2016, 16(7)867-75. 10.1016/j.spinee.2016.03.010 26970600

[41] Ruberté LM, Natarajan RN, Andersson GB (2009). Influence of single-level lumbar degenerative disc disease on the behavior of the adjacent segments—a finite element model study. J Biomech.

[42] Liang J, Dong Y, Zhao H (2014). Risk factors for predicting symptomatic adjacent segment degeneration requiring surgery in patients after posterior lumbar fusion. J Orthop Surg Res.

[43] Kim HJ, Kang KT, Chun HJ (2015). The influence of intrinsic disc degeneration of the adjacent segments on its stress distribution after one-level lumbar fusion. Eur Spine J.

